# The Aphid Genus *Lachnus* Burmeister, 1835 in Caucasus with Description of a New Species (Hemiptera, Aphididae, Lachninae) †

**DOI:** 10.3390/insects17100983

**Published:** 2026-09-23

**Authors:** Mariusz Kanturski, Shalva Barjadze

**Affiliations:** 1 Institute of Biology, Biotechnology and Environmental Protection, Faculty of Natural Sciences, University of Silesia in Katowice, ul. Bankowa 9, 40-007 Katowice, Poland; 2 Institute of Zoology, Ilia State University, Giorgi Tsereteli 3, 0162 Tbilisi, Georgia; shalva.barjadze@iliauni.edu.ge

**Keywords:** Aphidoidea, Lachnini, oak, *pallipes* species group, *Quercus*

## Abstract

The genus *Lachnus* Burmeister, 1835 (Hemiptera, Aphididae, Lachninae) comprises 35 valid species of medium to large-sized aphids associated with trees belonging to various plant families, mostly Fagaceae. This work reviews *Lachnus* species recorded so far in Transcaucasia (Georgia, Armenia, and Azerbaijan) and includes the description of a new species, *Lachnus caucasicus* **sp**. **nov**., belonging to the “*pallipes*” group, based on apterous and alate viviparous females collected in Georgia. The new species is compared with other congeners, focusing especially on morphological differences from *L*. *pallipes* (Hartig, 1841). Furthermore, updated keys to the species of the “*pallipes*” group, as well as to Caucasian *Lachnus* species and world *Quercus*-feeding species of *Lachnus*, are provided.

## Simple Summary

This paper presents a list of the aphid genus *Lachnus* Burmeister, 1835 (Hemiptera, Aphididae: Lachninae), in Transcaucasia that includes four species. Additional information and keys for identification of new species *L. caucasicus* **sp**. **nov**., *L. pallipes* Hartig, *L. roboris* (L.) and *L. wichmanni*, so far recorded in Armenia, Azerbaijan, and Georgia, are provided.

## 1. Introduction

The genus *Lachnus* Burmeister, 1835 (Hemiptera, Aphididae: Lachninae) comprises 35 valid species of which two are extinct [1]. Alive aphids belonging to this genus are known for their medium-to-large body size, are often dark brown or black, and can form large colonies that are often visited by ants [2,3]. *Lachnus* representatives are characteristic aphids, generally with long legs, having a body densely covered by setae, and from other genera of the tribe Lachnini Herrich-Schaeffer, 1854; they may be distinguished especially by their primary rhinaria with a sclerotic rosette, two to seven subapical setae on their processus terminalis, and characteristically pigmented wings of the alate viviparous females [3,4,5]. Species of the genus *Lachnus* are associated with trees from different plant families like Eleagnaceae, Fagaceae, Moraceae and Salicaceae, of which Fagaceae trees like *Castanea*, *Castanopsis*, *Fagus* and *Quercus* are the most preferable; *Lachnus* species may be found on their branches, bark or even main root [6,7]. In the not-so-distant past, the genus received some attention, e.g., Binazzi and Remaudière [7] described an unusual species in this genus—*L. sorini* Binazzi & Remaudière, with a characteristic dorsal abdominal tubercle. Later, the genus was reviewed by Binazzi and Remaudière [8], and part of *Lachnus* species, especially the so-called “*pallipes*” group, was revised by Kanturski et al. [4], who described a new species in this group—*L. pseudonudus* Kanturski & Wieczorek—and restored the species status of *L. chosoni* Szelegiewicz, treated as a synonym of *L. pallipes* by Blackman & Eastop [9]. Later, Mróz et al. [10] provided molecular analyses of some species of this group, undoubtedly listed *L. longirostris* as a synonym of *L. pallipes,* and designated a neotype for the latter. Recently, Kanturski et al. [11] described a new species, *L. margallaensis* Kanturski & Zia from Pakistan, related to *L. acutihirsutus* Kumar & Burghardt, indicating that a group of similar species in the “*acutihirsutus*” group may be distributed in Northern Pakistan and Northern India.

The “*pallipes*” group of the *Lachnus* species has been well-defined by Binazzi and Remaudière [8], first of all by the lack of mesosternal processes, although very flat and indistinct ones may be found in *L. chosoni*, so far known only from the Korean Peninsula [4,12,13]. Species like *L. crassicornis* Hille Ris Lambers, *L. chosoni*, *L. pseudonudus* and *L. pallipes* moreover share other characteristics that allow distinguishing them from other *Lachnus* members, namely the hind tibiae in which some of the setae on the dorsal inner side are much longer than others. They can be found throughout Europe and Caucasus (*L. pallipes*) [2,14] or in selected areas like Israel and Romania (*L. crassicornis*) [2,6,9], Türkiye (*L. pseudonudus*) [4], or the mentioned Korean Peninsula (*L. chosoni*) [13].

So far, 355 aphid species have been recorded from Georgia [15], of which two species belong to the genus *Lachnus*—*L. roboris* [15] and *L. wichmanni* Hille Ris Lambers [15,16].

An examination of the Aphididae collection at the Institute of Zoology, Ilia State University (Georgia), revealed specimens of a *Lachnus* species, previously identified as *L. pallipes*, that do not correspond with the morphological features of the *L. pallipes* neotype or its known descriptions [3,4,8,10]. This species can be distinguished from other members of the “*pallipes*” group by its leg pigmentation and several morphometric characteristics. Providing a formal description of this taxon is the main objective of the present study.

The following paper is a part of a revision of the genus *Lachnus*.

## 2. Materials and Methods

Slide specimens of apterous and known alate viviparous females used for this study were examined using a Leica DM 3000 LED light microscope and photographed using a Leica MC 190 HD camera (Leica Microsystems, Wetzlar, Germany) using a differential interference contrast (University of Silesia in Katowice, Poland). The measurements (including measurements of the first segment of the hind tarsus) were taken according to Ilharco and van Harten [17], Heie [3] and Kanturski et al. [4]. All of the measurements are given in millimeters. The following abbreviations are used: **ABD TERG**: abdominal tergite; **ANT**: antennae or their lengths; **ANT I–VI**: antennal segments I, II, III, IV, V, and VI or their lengths (ratios between antennal segments are simply given as, e.g., “VI: III”); **BASE**: basal part of the last antennal segment or its length; **BD III**: basal articular diameter of ANT III; **BL**: body length (from the anterior border of the head to the end of cauda); **BW**: maximal width of body; **HT I**: first segment of the hind tarsus; **HT II**: second segment of the hind tarsus or its length; **HW**: greatest head width across the compound eyes; **LS ANT III**: length of the longest setae of ANT III; **PT**: processus terminalis of the last antennal segment or its length; **SIPH**: siphunculi or its diameter; **URS**: ultimate segments of the rostrum (IV + V) or their lengths; **III FEMORA**: hind femora length; and **III TIBIAE**: hind tibiae length. In the case of a series of single slides with a single specimen with the same collection data for the examined material sections, all of them present the same data as the full previous slide in order to avoid repetition.

The holotype and paratypes of the new species will be deposited in **IZISU**—the Institute of Zoology, Ilia State University, Tbilisi (Georgia). One paratype will be additionally deposited in **DZUS**—Zoology Research Team, University of Silesia, in Katowice Hemiptera Collection, Katowice (Poland).

## 3. Results

### 3.1. Lachnus of Transcaucasia

*Lachnus* Burmeister, 1835

*Lachnus caucasicus* **sp**. **nov**.

urn:lsid:zoobank.org:act:345BBD88-9C33-407C-AAAF-9B6E07FA3770

 

**Description**

*Apterous viviparous female*

Color in life: unknown. Pigmentation of mounted specimens: Body, in general, light brown to brown. Head, pronotum and mesonotum brown. ANT light brown with brown distal parts of ANT II–IV and with brown ANT V and VI with slightly lighter basal parts. Fore femora uniformly brown, middle femora with a central, outer brown patch, hind femora brown with light-brown proximal and distal parts. Fore and middle tibiae light brown with a delicate darker patch near the proximal end and brown distal ends. Hind tibiae predominantly brown with evidently darker distal ends and very poorly visible slightly darker distal inner patch. Tarsi brown. Abdomen light brown with brown sclerites, intersegmental muscle sclerites, light-brown SIPH and brown genital plate, anal plate and cauda (Figure 1a and Figure 2a,b,e).

**Figure 1:**
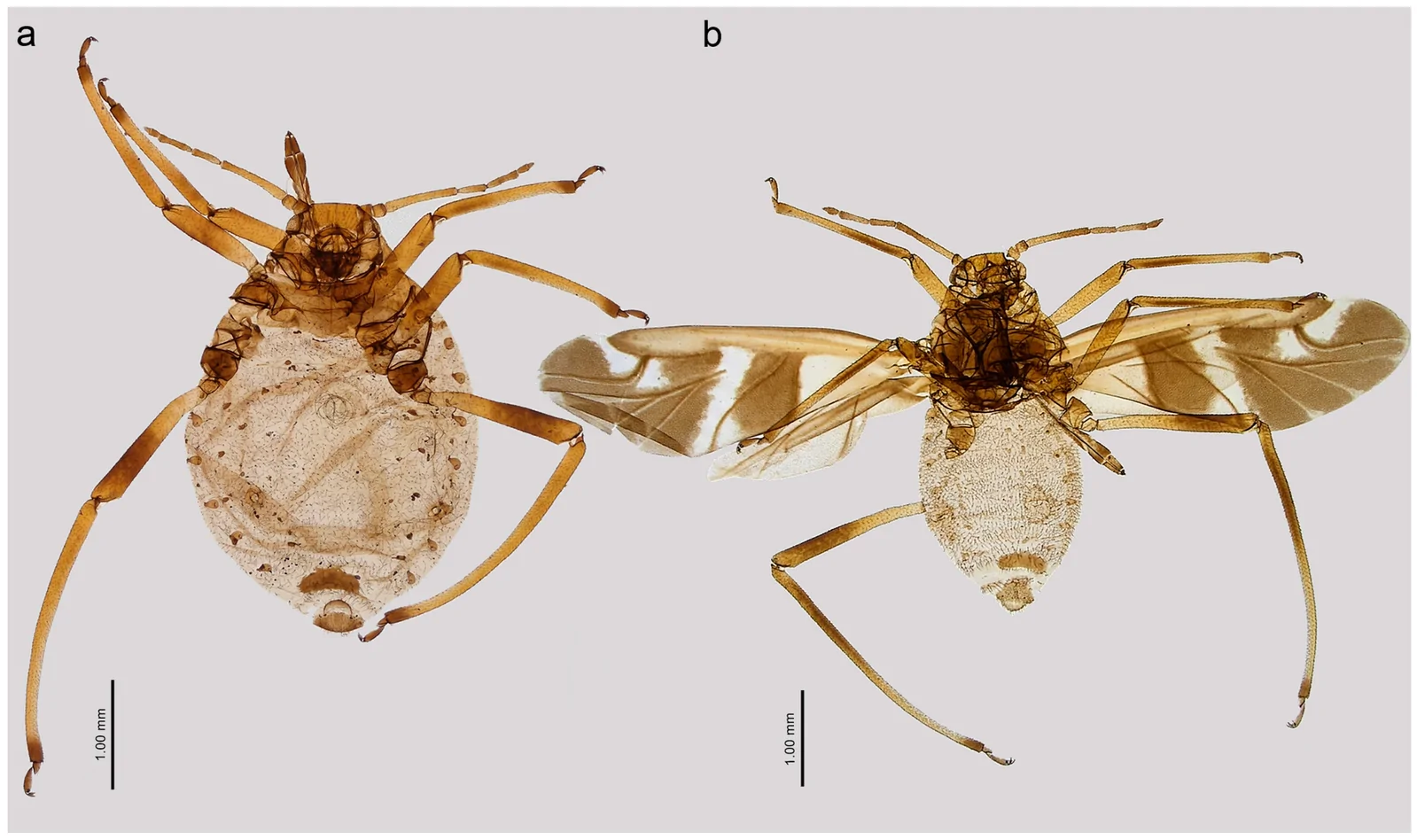
*Lachnus caucasicus* sp. nov. habitus of (**a**) apterous viviparous female and (**b**) alate viviparous female.

Body egg-shaped (Figure 1a). Head densely covered by pigmented, long, and fine setae, with extremely fine apices. Frons flat (Figure 2b). HW 0.50–0.52 × ANT. ANT 6-segmented (Figure 2a), 0.39–0.42 × BL. ANT III shorter than ANT IV + V + VI, with 1 small and poorly visible secondary rhinarium. ANT IV shorter than ANT V, with 0–1 small secondary rhinarium. ANT V longer than ANT VI. ANT VI with PT 0.29–0.44 × BASE, with 16–18 basal, 3–4 apical and 3–4 subapical setae (Figure 2c). Other antennal ratios: VI:III 0.23–0.26; V:III 0.45–0.51; IV:III 0.37–0.41; IV:V 0.77–0.84. ANT I–VI densely covered with pigmented, long, fine setae with very fine pointed apices, 0.050–0.085 mm long, LS BD III 0.97–1.13 × BD III. Rostrum, reaching hind coxae. URS tapering, pointed, 0.32–0.33 × ANT III, 1.28–1.40 × ANT VI and 0.76–0.90 × HT II, with 23–25 accessory setae (Figure 2d). Mesosternal tubercles absent (Figure 2b). Hind legs 0.99–1.12 × BL, covered by pigmented, long setae with very fine apices, 0.04–0.08 mm long on III TIBIAE and 0.05–0.085 on III TIBIAE. Some setae on the ventral side of hind tibiae are about 2–3 times longer than other ones, 0.11–0.14 mm long (Figure 2f). HT I basal length 1.48–1.75 × dorsal, 0.46–0.50 × ventral and 0.62–0.70 × intersegmental length, with 12–14 ventral and two dorso-lateral setae (Figure 2g). HT II 0.37–0.44 × ANT III and 1.42–1.82 × ANT VI. Abdomen membranous, cuticle with slightly wrinkled or ruffled surface (Figure 2h). SIPH sclerites small but well-developed, usually rounded or slightly oval, with smooth edges (Figure 2i). Abdomen covered by long, pigmented and pointed setae with very fine apices, 0.08–0.10 mm long on ABD I-V and 0.07–0.11 mm long on ABD VI–VIII (Figure 2h,i). ABD VIII with 42–77 setae, 0.06–0.11 mm long. Subgenital plate well-developed, with a little indentation from the posterior side. Cauda circular or semicircular, anal plate well-developed, sclerotized.

**Figure 2:**
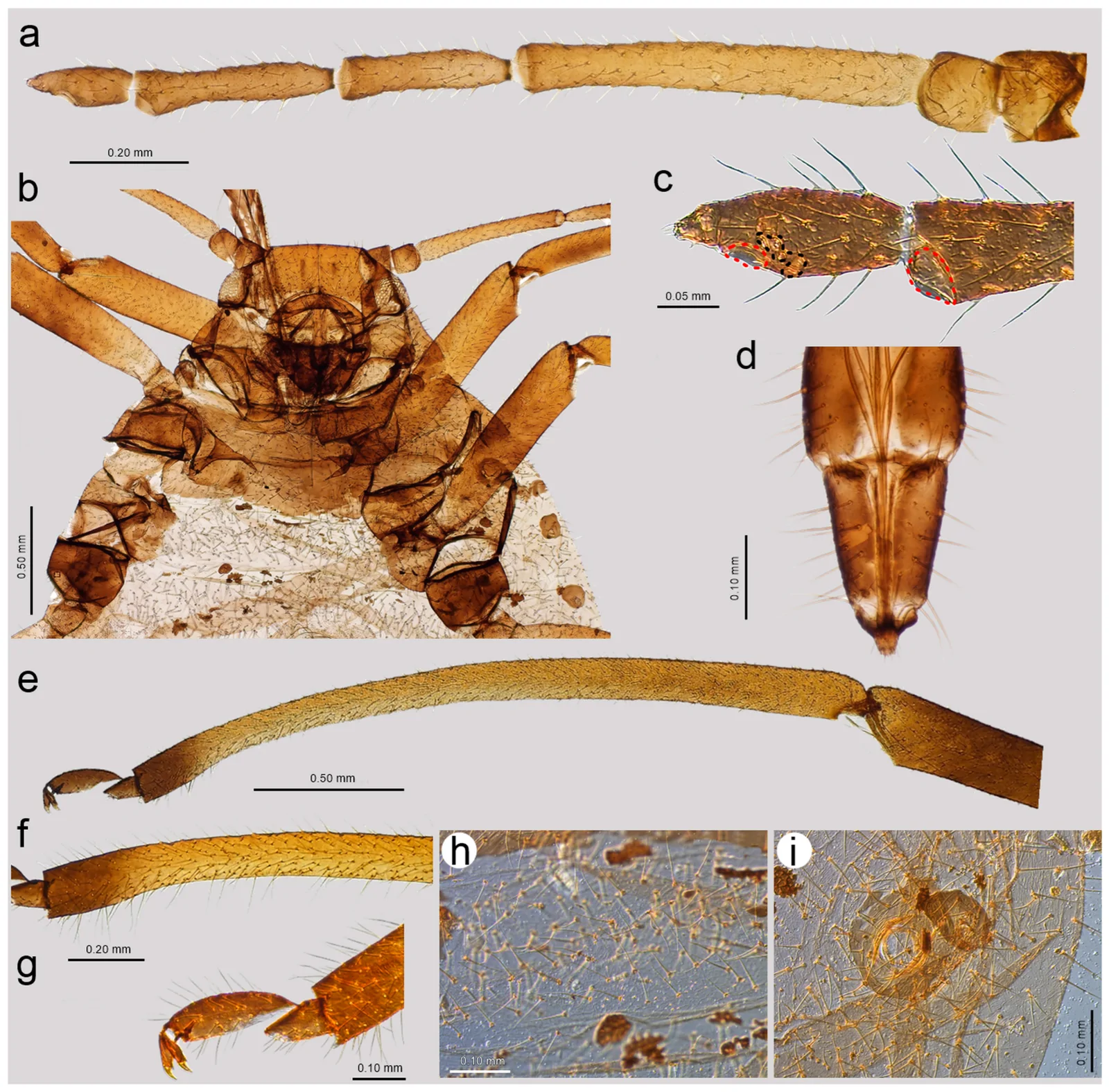
*Lachnus caucasicus* sp. nov. apterous viviparous female—detailed morphological characteristics: (**a**) antenna, (**b**) head, thorax and anterior part of abdomen, (**c**) ANT V and VI, with primary rhinarium on ANT V and major rhinarium on ANT VI (red dotted circle) and accessory rhinaria (black dotted line), (**d**) ultimate rostral segments, (**e**) hind leg, (**f**) end of hind tibia with extra-long setae, (**g**) hind tarsus, (**h**) dorsal abdominal cuticle and chaetotaxy, (**i**) SIPH.

*Alate viviparous female*

Color in life: unknown. Pigmentation of mounted specimens: Body in general light brown to brown. Head, pronotum and thorax sclerotized, brown. ANT light brown with brown distal parts of ANT II–V and with brown ANT VI. Fore wings infuscated from Cu1b with a middle, colorless band, colorless patch between the pterostigma and Rs, and patch in the pterostigma, R and second media bifurcation. The very basal part of the wing slightly dusky. Fore and middle femora uniformly brown, hind femora brown with light-brown proximal half. Fore and middle tibiae light brown with darker longitudinal stripe patch on the inner proximal part and brown distal end. Hind femora light brown with brown proximal part (the knee area paler) and brown distal end. Tarsi brown. Abdomen pale brown with brown sclerites and intersegmental muscle sclerites, light-brown SIPH and brown genital plate, anal plate and cauda (Figure 1b and Figure 3a,e,g).

**Figure 3:**
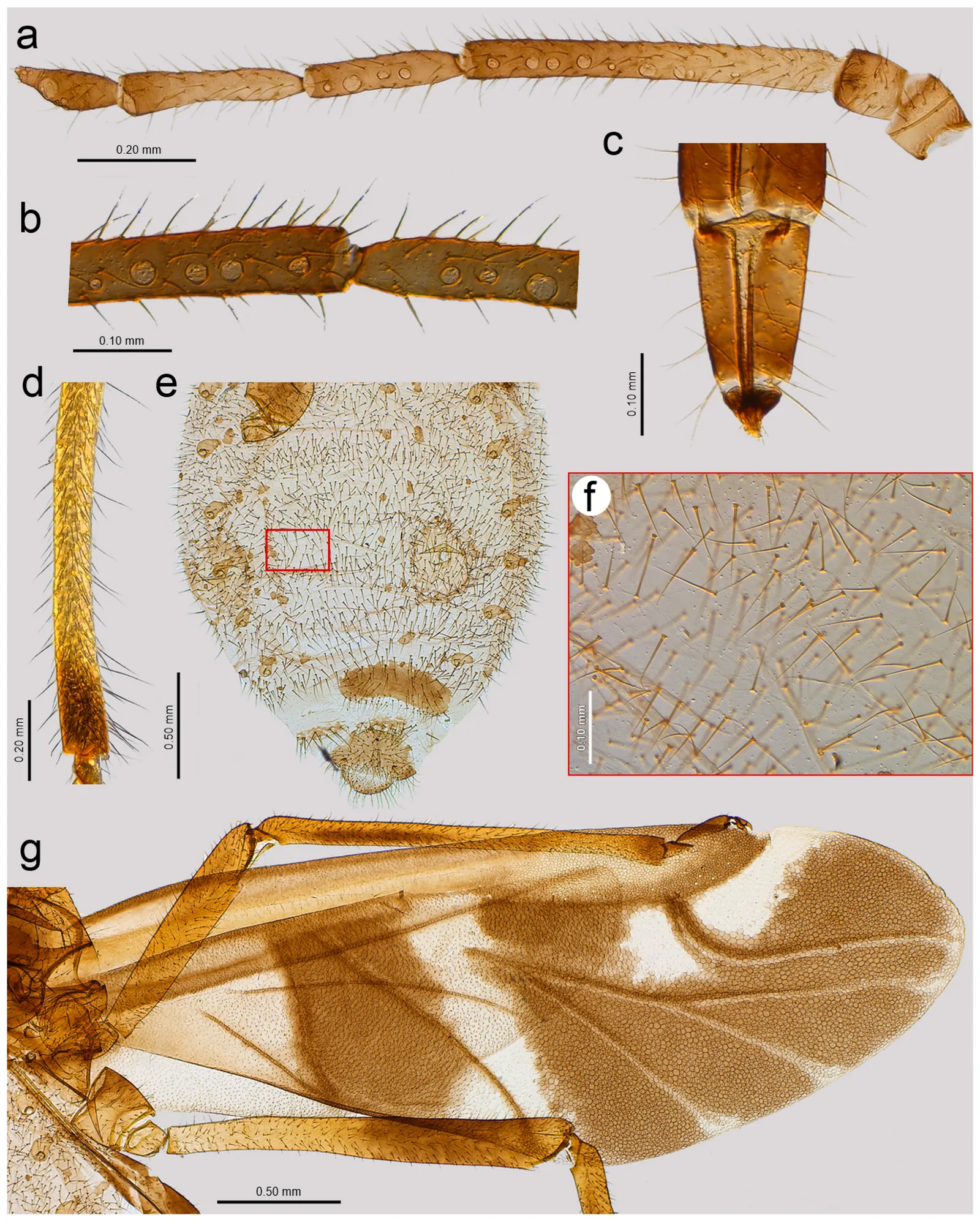
*Lachnus caucasicus* sp. nov. alate viviparous female—detailed morphological characteristics: (**a**) antenna, (**b**) secondary rhinaria on ANT III and IV, (**c**) ultimate rostral segments, (**d**) end of hind tibia with extra-long setae, (**e**) abdominal sclerotization and chaetotaxy, (**f**) dorsal abdominal cuticle and chaetotaxy, (**g**) fore-wing pigmentation.

Body oval (Figure 1b). Head densely covered by pigmented, long, and fine setae, with extremely fine apices. Frons flat. HW 0.41–0.49 × ANT. ANT 6-segmented (Figure 3a), 0.42–0.50 × BL. ANT III shorter than ANT IV + V + VI, with 8–11 rounded and flat secondary rhinaria of different sizes in one line reaching almost the whole length of the segment (Figure 3a,b). ANT IV shorter than ANT V, with 3–5 secondary rhinaria of the same characters as on ANT III (Figure 3b). ANT V longer than ANT VI. ANT VI with PT 0.26–0.40 × BASE, with 17–20 basal, 3 apical and 5 subapical setae. Other antennal ratios: VI:III 0.25–0.32; V:III 0.47–0.55; IV:III 0.40–0.42; IV:V 0.77–0.84. ANT I–VI densely covered with pigmented, long, fine setae with very fine pointed apices, 0.050–0.090 mm long, LS BD III 1.14–1.38 × BD III. Rostrum, reaching SIPH. URS tapering, pointed, 0.35–0.40 × ANT III, 1.27–1.42 × ANT VI and 0.90–0.94 × HT II, with 28–30 accessory setae (Figure 3c). Thorax sclerotized, fore wings with media twice-branched, hind wings with two oblique veins (Figure 3g). Hind legs 1.35–1.50 × BL, covered by pigmented, long setae with very fine apices, 0.03–0.08 mm long on III TIBIAE and 0.05–0.080 on III TIBIAE. Some setae on the ventral side of hind tibiae are about 2–3 times longer than other ones, at 0.11–0.15 mm long (Figure 3d). HT I basal length 1.10–1.20 × dorsal, 0.37–0.43 × ventral and 0.58–0.69 × intersegmental length, with 14 ventral and two dorso-lateral setae. HT II 0.38–0.42 × ANT III and 1.34–1.57 × ANT VI. Abdomen membranous, cuticle with very slightly wrinkled surface (Figure 3e,f). SIPH sclerites well-developed, oval, with smooth or slightly jagged edges, especially in the distal end. Abdomen covered by long, pigmented and pointed setae with very fine apices, 0.075–0.085 mm long on ABD I–V and 0.075–0.090 mm long on ABD VI–VIII (Figure 2f). ABD VIII with 24–30 setae, 0.10–0.15 mm long. Subgenital plate well-developed, with very slight indentation from the posterior side. Cauda circular or semicircular, anal plate well-developed, sclerotized.

Detailed measurements values are given in Table 1.

 

**Type material examined**: Holotype. Apterous viviparous female (apt.), GEORGIA, Mtskheta-Mtianeti region, Mtskheta municipality, Tsodoreti village, 900 m., 01.06.1965, *Quercus petraea* subsp. *iberica* (Steven ex M.Bieb.) Krassiln. (on slide as oak in Cyrillic) A. Dzhibladze leg., IZISU. Paratypes. 1 Apt., 1 al. other data same as in holotype, IZISU; 1 al. other data same as in holotype, DZUS.

**Note**: The second author found A. Dzhibladze’s unpublished manuscript typed in Georgian on a typewriter. In the manuscript, she wrote under the *Lachnus pallipes*: “it is mentioned on the twigs of oak (*Quercus iberica*). Tsodoreti, 1.6.1965”. For the slides with aphids erroneously determined as *Lachnus pallipes* by Dzhibladze, the same sampling locality and date are written by her in Cyrillic. Only the host plant is written ––as oak in Cyrillic without a suitable Latin name. *Quercus iberica* now has a subspecies status as *Quercus petraea* subsp. *iberica* (Steven ex M.Bieb.) Krassiln [18].

 

**Diagnosis**: The new species, due to the hind tibiae ventral setae dimorphism, should be recognized as a new one in the so-called “*pallipes*” group of species [4]. Due to the pigmentation of the hind tibiae (predominantly brown with eventually darker spots in the proximal part and a darker distal end), *L. caucasicus* can be easily distinguished from *L. crassicornis*, *L. pallipes* and *L. pseudonudus* (in all species, the hind tibiae are dark in the proximal and distal parts with a lighter middle part). Additionally, the new species is larger (4.10–4.34 mm) than *L. crassicornis* and *L. pseudonudus* (2.25–2.52 mm and 2.62–3.30, respectively). According to the pigmentation of the hind tibiae, *L. caucasicus* is similar to *L. chosoni*, from which it can be distinguished by the absence of mesosternal tubercles (little ones in *L. chosoni*) and a lower ratio of ANT IV/ANT V—0.77–0.85 (1.00–1.10 in *L. chosoni*). General differences between the new species within the “*pallipes*” group are presented in Table 2.

As *L. pallipes* is the most common species from this morphologically similar group, we would like to show more differences between the latter and the new species. *Lachnus caucasicus* differs from *L. pallipes*, first of all, in the following:

- Lower ratio of URS/HT II—0.77–0.90 (0.96–1.00 in *L. pallipes*);
- Higher ratio of HW/ANT—0.51–0.52 (0.43–0.46 in *L. pallipes*);
- Higher ratio of HT II/ANT VI—1.43–1.53 (1.32–1.39 in *L. pallipes*);
- Lower ratio of ANT/BL—0.39–0.42 (0.44–0.49 in *L. pallipes*);
- Higher ratio of ANT V/ANT III—0.46–0.51 (0.40–0.44 in *L. pallipes*);
- Higher ratio of HTI basal/HT I dorsal length—1.49–1.75 (0.69–1.10 in *L. pallipes*);
- Higher ratio of HT I basal/HT I ventral length—0.46–0.50 (0.33–0.41 in *L. pallipes*).

 

**Etymology**: The specific name of the new species “*caucasicus*” is a Latinized adjective and means “of the Caucasus” or “Caucasian”.

 

**Host plant**: This species was collected on the twigs of *Quercus petraea* subsp. *iberica* (Steven ex M.Bieb.) Krassiln.

 

*Lachnus pallipes* (Hartig, 1841)

 *Pterochlorus roboris* var. *fagarum* (exsiccator Alt?) [14]

 

A doubtful record of this species on oak was reported from the Shamakhi district in Azerbaijan [14]. *Lachnus pallipes* has never been recorded in Armenia or Georgia [14,19]. Although the occurrence of *L. pallipes* in the Caucasus has been mentioned in the literature [2,6,9], these references most likely originate from the report by Rusanova [14].

 

*Lachnus roboris* (Linnaeus, 1758)

 

It is a widely distributed in Armenia, Azerbaijan and Georgia [14,15,19].

 

*Lachnus wichmanni* Hille Ris Lambers, 1956

 

This species was found in two localities of Southern Georgia: in Akhaltsikhe and Kvabiskhevi, in the Samtskhe-Javakheti region, as ants attended dense colonies on the trunk and branches of *Hippophae rhamnoides* L. in July 2009 [16]. All attempts to sample this aphid species in the same or other localities in Georgia were unsuccessful. It seems that it is a rare species. In the Caucasus, it is known only from Georgia.

 

### 3.2. Identification Keys

Key to Caucasian *Lachnus* species with *L. pallipes* include the following:

 

**1.**  Hind tibiae with some more distal setae on ventral side, long and fine-pointed and at least 2–3 × longer than the neighboring short setae. Mesosternum without tubercles or processes … **2**
  - Hind tibiae with setae on ventral side often longer and finer distally, but with no hairs 2–3 × longer than others. Mesosternum with tubercles or processes … **3**
**2.**  Hind tibiae predominantly brown with eventually darker spots in the proximal part and a darker distal end. URS/HT II 0.76–0.90, HTI basal/HT I dorsal length—1.49–1.75 … ***Lachnus caucasicus*** sp. nov.
  - Hind tibiae dark in proximal and distal parts with lighter middle part. URS/HT II 0.96–1.00, HTI basal/HT I dorsal length—0.69–1.10 … ***Lachnus pallipes*** Hartig
**3.**  Mesosternal processes short and cup-shaped, head with membranous wedge … ***Lachnus wichmanni*** Hille Ris Lambers
  - Mesosternal processes longer and finger-shaped, head without membranous wedge … ***Lachnus roboris*** (L.)

 

Updated key of Kanturski et al. [4] to apterous viviparous females of the “*pallipes*” group of *Lachnus* species:

 

**1.**  Siphunculi almost poriform with almost invisible sclerites. Dorsum covered with thin, long, hair-like and pointed setae, which are almost similar to ventral setae … ***Lachnus crassicornis*** Hille Ris Lambers
  - Siphunculi with well-developed sclerites. Dorsal setae variable but always different from setae on ventral side … **2**
**2.**  Dorsal abdominal setae short and inconspicuous (max 0.02 mm) with blunt, capitate or club-shaped apices, and arising from large, ring-like bases … ***Lachnus pseudonudus*** Kanturski & Wieczorek
  - Setae on dorsal side of abdomen always longer than 0.037 mm and pointed, not arising from large, ring-like bases … **3**
**3.**  HT II/ANT VI 1.43–1.53; ANT/BL 0.39–0.42; ANT V/ANT III 0.46–0.51 … ***L. caucasicus*** sp. nov.
  - HT II/ANT VI 1.20–1.39; ANT/BL 0.44–0.50; ANT V/ANT III 0.35–0.44 … **4**
**4.**  Hind tibiae brown on distal and proximal parts with pale area for about 1/3 of length. ANT IV/ANT V 0.78–0.96, URS/ANT VI 1.26–1.38, URS/HT II 0.96–1.00 … ***Lachnus pallipes*** Hartig
  - Hind tibiae with distal part uniformly dark brown and proximal part paler. ANT IV/ANT V 1.00–1.10, URS/ANT VI 1.00–1.06, URS/HT II 0.79–0.84 … ***Lachnus chosoni*** Szelegiewicz

 

Updated Blackman and Eastop’s key D—Lachninae on oaks (from couplet No. 13):

 

**13.**  Hind tibia with some more distal hairs on ventral side, very long and fine-pointed and at least 2–3 × longer than neighboring short hairs (as in Figure 93D) … **14**
  - Hind tibia with hairs on ventral side often longer and finer distally, but with no hairs 2–3 × longer than others … **17** (changed to the old couplet 16)
**14.**  Dorsal body hairs short (less than 30 µm), with blunt or capitate apices, arising from large, ring-like bases … ***Lachnus pseudonudus***
  - Dorsal body hairs long and fine-pointed (more than 35 µm), with pointed apices … **15**
**15.**  HT II/ANT VI 1.43–1.53; ANT/BL 0.39–0.42; ANT V/ANT III 0.46–0.51 … ***Lachnus caucasicus*** sp. nov.
  - HT II/ANT VI 1.20–1.39; ANT/BL 0.44–0.50; ANT V/ANT III 0.35–0.44 … **16**
**16.**  Hind tibiae with proximal half mainly dark except at base, and distal part somewhat paler except near apex. ANT VI 0.14–0.19 mm long (BASE 0.103–0.138 mm, PT 0.035–0.058 mm). Mesosternal processes absent … ***Lachnus pallipes***
  - Hind tibiae with basal half paler and distal part uniformly dark brown. ANT VI 0.21–0.25 mm long (BASE 0.140–0.170 mm, PT 0.070–0.080 mm). Mesosternum with a pair of small, flat processes … ***Lachnus chosoni***
**17.**  SIPH cones small and pale (basal diameter 0.09–0.13 mm). Hairs on ABD TERG 1–6 all short and blunt, maximally about 10 µm … ***Lachnus tuatayae***
  - SIPH cones usually large (basal diameter more than 0.3 mm) and well-pigmented. Hairs on ABD TERG 1–6 variable, but the longest always more than 20 µm … **18**

## 4. Discussion

Creating and proposing (even operational) species groups for morphologically diverse species within a given genus is a useful procedure in taxonomic research, not only within Lachninae [4,8,11] but also in other subfamilies, such as Drepanosiphinae [20]. The Fagaceae-feeding species of the genus *Lachnus* constitute a morphologically similar and widely distributed group [8]. Within the genus, *Quercus*-feeding representatives are divided into three main lineages: the western Palaearctic “*roboris*” and the eastern Palaearctic-Oriental “*acutihirsutus*” and “*tropicalis*” groups [8,11]. Among the western representatives, the “pallipes” group represents a distinct cluster characterized by the lack (or indistinct presence) of mesosternal tubercles in apterous viviparous females and the characteristic setae diversity on the hind tibiae, where some ventral setae are distinctly longer than the others in all morphs [4,8,13].

The discovery of *Lachnus caucasicus* in Georgia introduces a new, well-defined taxon within the “*pallipes*” group. Morphologically, *L. caucasicus* shares the characteristic presence of much longer fine and pointed ventral setae on hind tibiae with *L. chosoni*, *L. crassicornis*, *L. pallipes* and *L. pseudonudus*. Also, the lack of mesosternal tubercles directly assigns the new species to the “*pallipes*” group, separating it from other Palearctic and Oriental taxa (such as *L. acutihirsutus*, *L. roboris*, *L. takahashii* Sorin and *L. shiicola* Sorin) that bear tapered or mammiform projections [2,3,8]. However, the predominantly brown pigmentation of its hind tibiae (with paler proximal and darker distal ends) helps to easily distinguish it from *L. crassicornis*, *L. pallipes* and *L. pseudonudus*, all of which are characterized by the pale area in the middle of hind tibiae. Although this specific hind tibiae pigmentation pattern resembles that of the eastern Palearctic species *L. chosoni*, the newly described one clearly differs from it by the absence of mesosternal tubercles (which are present, though flat and small, in *L. chosoni*) and significantly different antennal ratios, like the much lower ANT IV/ANT V ratio (0.77–0.85 in *L. caucasicus* vs. 1.00–1.10 in *L. chosoni*). Furthermore, *L. caucasicus* is characterized by larger body length (4.10–4.34 mm) than *L. crassicornis* (2.25–2.52 mm) and *L. pseudonudus* (2.62–3.60 mm). Due to its general morphology, *L. caucasicus* could be confused with *L. pallipes*, the most common and widely distributed representative of the species with extra-long hind-tibial setae. Although both species reach a similar overall body length, the new one is distinctly more massive. The maximum body width of *L. caucasicus* (2.77–2.80 mm) clearly exceeds that of *L. pallipes* (2.00–2.50 mm). This robustness is also evident in head width, which is wider in the new species (0.88–0.91 mm) than in *L. pallipes* (0.79–0.85 mm). Interestingly, despite its larger body, *L. caucasicus* has shorter antennae (1.71–1.75 mm) than *L. pallipes* (1.81–1.92 mm). This character is also noticeable in the length of ANT III (0.67–0.70 mm vs. 0.75–0.82 mm). These differences between diagnostic morphometric ratios clearly separate these two taxa, with no overlap in their ranges. First of all, the HW/ANT ratio in *L. caucasicus* is 0.51–0.52, whereas in *L. pallipes* it does not exceed 0.46. The apical segments of the rostrum are proportionally much shorter than the second segment of the hind tarsus (URS/HT II is 0.77–0.90 in *L. caucasicus* compared to 0.96–1.00 in *L. pallipes*). Additional distinguishing features between the two species can also be identified by analyzing the length of the first segment of the hind tarsus (HT I). The basal part of HT I in the new species is distinctly longer (0.060–0.070 mm) than in *L. pallipes* (0.045–0.055 mm). As a result, the ratio of the HT I basal length to the HT I dorsal length ranges from 1.49 to 1.75 in *L. caucasicus*. In strong contrast, both lengths are nearly equal in L. pallipes, or the dorsal margin is slightly longer, resulting in a basal/dorsal ratio of only 0.69–1.10. Additionally, the basal/ventral length ratio of HT I is higher in the new species (0.46–0.50) compared to *L. pallipes* (0.33–0.41). Differences in the number of secondary rhinaria and ANT VI chaetotaxy further support the separation of the new species. Apterous viviparous females of *Lachnus caucasicus* exhibit a low number of secondary rhinaria on ANT III (consistently 1 rhinarium) and subapical setae on the PT (3–4 setae), compared to the more variable and higher numbers found in *L. pallipes* (2–6 secondary rhinaria on ANT III and PT with 5–6 subapical setae).

From a zoogeographical perspective, both *L. crassicornis* and *L. pseudonudus* have been recorded from very restricted Mediterranean or Sub-Mediterranean localities like Israel, Romania and Türkiye, while *L. chosoni* is still known only from the Korean Peninsula. The presence of *L. caucasicus* in the higher-altitude region of Georgia provides new data for future understanding of the “*pallipes*” group and bridges the gap between western and eastern Palearctic species.

And finally, while the presence of distinctly elongated ventral setae on the hind tibiae, combined with the absence of mesosternal tubercles, successfully delineates the “*pallipes*” group morphologically, comprehensive future molecular phylogenetic studies are needed. Such analyses will be crucial to definitively determine whether this unique chaetotaxy reflects true monophyly among these widespread taxa or has emerged independently through convergent evolution driven by specific ecological or behavioral adaptations.

## Figures and Tables

**Table 1:** Measurements of apterous and alate viviparous females of *L. caucasicus* sp. nov.

Character	Apterous Viviparous Female	Alate Viviparous Female
**BL**	4.100–4.340	3.300–3.870
**BW**	2.770–2.800	1.680–1.800
**HW**	0.880–0.910	0.700–0.810
**ANT**	1.700–1.750	1.635–1.705
**ANT III**	0.250–0.280	0.630–0.700
**ANT IV**	0.260–0.280	0.270–0.280
**ANT V**	0.320–0.350	0.320–0.350
**ANT VI**	0.175–0.189	0.175–0.189
**BASE**	0.110–1.135	0.125–0.160
**PT**	0.040–0.079	0.041–0.050
**URS**	0.223–0.225	0.250–0.256
**III FEMORA**	1.470–1.530	1.580–1.750
**III TIBIAE**	2.200–2.750	2.950–3.05
**HT I basal**	0.060–0.070	0.050–0.055
**HT I dorsal**	0.040–0.045	0.043–0.050
**HT I ventral**	0.130–0.140	0.120–0.135
**HT I intersegmental**	0.095–0.108	0.075–0.085
**HT II**	0.250–0.290	0.270–0.275
**SIPH**	0.170–0.180	0.360

**Table 2:** General morphometric differences between apterous viviparous females of species of the “*pallipes*” group.

Character	*Lachnus* *caucasicus*	*Lachnus* *crassicornis*	*Lachnus* *pseudonudus*	*Lachnus* *chosoni*	*Lachnus* *pallipes*
**BL**	4.10–4.34	2.25–2.52	2.62–3.60	3.80–4.20	3.90–4.12
**HW**	0.88–0.91	0.60–0.70	0.68–0.81	0.85–0.87	0.79–0.85
**ANT/BL**	0.39–0.42	0.58–0.64	0.44–0.52	0.47–0.50	0.44–0.49
**URS/HT II**	0.77–0.90	1.00–1.20	1.00–1.19	0.79–0.85	0.96–1.00
**HT II/ANT VI**	1.43–1.53	1.33–1.73	1.38–1.61	1.20–1.35	1.32–1.39
**ANT V/ANT III**	0.46–0.51	0.37–0.45	0.44–0.54	0.35–0.41	0.40–0.44
**ANT IV/ANT V**	0.77–0.85	0.85–0.96	0.71–0.86	1.00–1.10	0.79–0.97

## Data Availability

All data generated or analyzed during this study are included in this published article.
